# Aspects électrocardiographiques de jeunes basketteurs sénégalais

**DOI:** 10.11604/pamj.2020.35.4.14812

**Published:** 2020-01-09

**Authors:** Désiré Alain Affangla, Mohamed Leye, Angèle Wabo Simo, El Hadji Mamadou Ndiaye, Franck D'Almeida, Thérèse Yandé Sarr, Abdoul Kane

**Affiliations:** 1 UFR Sciences de la Santé de l’Université de Thiès, Sénégal; 2 Hôpital Saint Jean de Dieu de Thiès, Sénégal; 3 Centre Hospitalier Régional de Thiès, Sénégal

**Keywords:** Electrocardiogram (ECG), sport, basketball, black, Senegalese, Electrocardiogramme (ECG), sport, basket, noir, Sénégalais

## Abstract

La pratique intensive et prolongée de sport peut induire des modifications cardiovasculaires et électrocardiographiques. L’objectif de ce travail était de décrire les modifications électrocardiographiques attribuables à la pratique de basket de compétition chez de jeunes noirs sénégalais. Les auteurs ont réalisé une étude prospective descriptive de l’électrocardiogramme (ECG) de surface de jeunes noirs sénégalais pratiquant le basket de compétition. Il s’agissait de 40 jeunes basketteurs, 20 filles et 20 garçons, dont la moyenne d’âge était respectivement de 17 ans ± 0,86 (extrêmes: 17 et 19 ans) et 15 ans ± 1,56 (extrêmes: 13 et 18 ans). La fréquence cardiaque était plus basse chez les garçons, 59 battements ± 9 battements (extrêmes: 42 et 85) que chez les filles 73 battements / min ± 11 battements (extrêmes: 50-95) (p = 0,0004). Les particularités suivantes ont été observées: des troubles de la repolarisation à type d’onde T négative de V1 à V4 dans 3 cas (7, 5%), une hypertrophie ventriculaire droite dans 1 cas (2,5%), une déviation axiale droite de QRS dans 1 cas (2,5%). La pratique intense et prolongée de basket entraîne des modifications électrocardiographiques chez le jeune noir sénégalais.

## Abstract (en)

Intensive and prolonged practice of sport can lead to cardiovascular and electrocardiographic changes. The purpose of this study was to describe the electrocardiographic changes in some young black Senegalese players practicing competitive basketball. We conducted a prospective descriptive analysis of surface electrocardiogram (ECG) findings related to young Senegalese Black players practicing competitive basketball. The study involved 40 young basketball players, 20 girls and 20 boys, whose average age was 17 ± 0.86 years (ranging from 17 to 19 years) and 15 ± 1.56 years (ranging from 13 to 18 years) respectively. Heart rate was lower among boys, 59 beats ± 9 beats (ranging from 42 to 85) than among girls 73 beats/ min ± 11 beats (ranging from 50 to 95) (p = 0.0004). The following features have been observed: repolarization abnormalities such as T-wave inversion V1-V4 in 3 cases (7.5%), right ventricular hypertrophy in 1 case (2.5%), right axis deviation (QRS axis) in 1 case (2.5%). Intensive and prolonged practice of basketball leads to electrocardiographic changes in the young black Senegalese players.

## Introduction

La pratique régulière et intensive de sport et particulièrement le sport de compétition peut induire des modifications cardiovasculaires observables à l’électrocardiographie [1]. Ces modifications peuvent parfois simuler à s’y méprendre une pathologie et amener à tort ne pas signer le certificat de non contre-indication à la pratique de sport de compétition compte tenu du spectre de mort subite auquel le sportif de compétition est exposé [2, 3]. L’objectif de ce travail était de décrire les modifications électrocardiographiques observées chez de jeunes basketteurs de compétition noirs sénégalais.

## Méthodes

Il s’agit d’une étude prospective portant sur l’analyse de l’électrocardiogramme de jeunes noirs sénégalais pratiquant le basket de compétition. Ces jeunes sportifs pratiquaient plus de 6 heures d’entraînement par semaine depuis plus d’un an. Ils ont bénéficié chacun d’un examen clinique et d’un ECG de repos avec un appareil Schiller AT1. L’ECG était enregistré par le même manipulateur rigoureusement de la même manière notamment avec les électrodes précordiales aux mêmes emplacements chez tous les sujets. Tous les ECG étaient interprétés par un cardiologue. Tous les sujets qui présentaient une anomalie à l’ECG ont bénéficié d’une exploration complémentaire par échographie Döppler cardiaque avec un appareil Myndray DC7. Le recueil des données et l’analyse statistique ont été effectués par le logiciel Excel. Le test de Student est utilisé pour comparer les moyennes et le Chi2 pour les pourcentages. Le seuil de signification a été fixé à 5%.

## Résultats

Il s’agissait de 40 basketteurs, 20 garçons et 20 filles. Leur moyenne d’âge était de 15,8 ans ± 1,56 (extrêmes: 13 et 18 ans) chez les garçons et de 17,60 ans ± 0,86 (extrêmes: 17 et 19 ans) chez les filles. Nous n’avons relevé aucun antécédent familial de mort subite ou d’évènement cardiovasculaire précoce. L’examen clinique était normal chez tous les sujets.

L’ECG a révélé: une fréquence cardiaque moyenne de 59 battement /min ± 9 battements (extrêmes: 42 et 85) chez les garçons et de 73 battements / min ± 11 battements (extrêmes: 50 et 95) chez les filles. La fréquence cardiaque au repos était plus basse chez les garçons que chez les filles (p = 0.0004); la durée moyenne de l’intervalle PR était de 0,17 sec ± 0,026 chez les garçons et 0,16 sec ±0,017 chez les sportives; la durée moyenne de l’intervalle QT corrigé mesurée manuellement était normale à 0,38 sec ± 0,010 chez les garçons et chez les filles.

**Figure 1:**
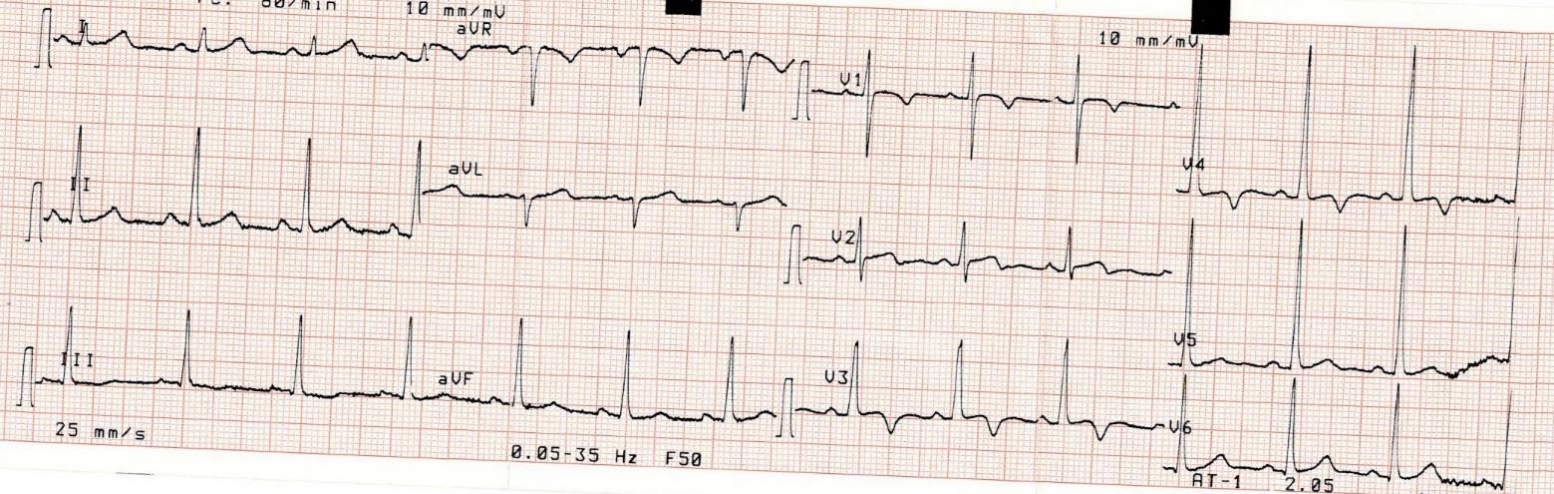
Onde T négative de V1 à V4

**Figure 2:**
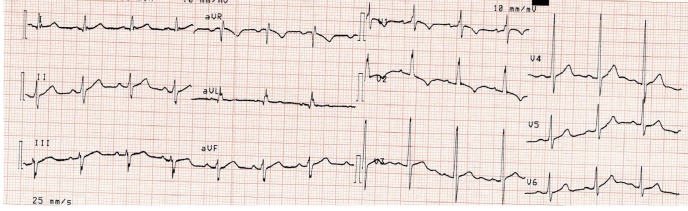
Hypertrophie ventriculaire droite

**Figure 3:**
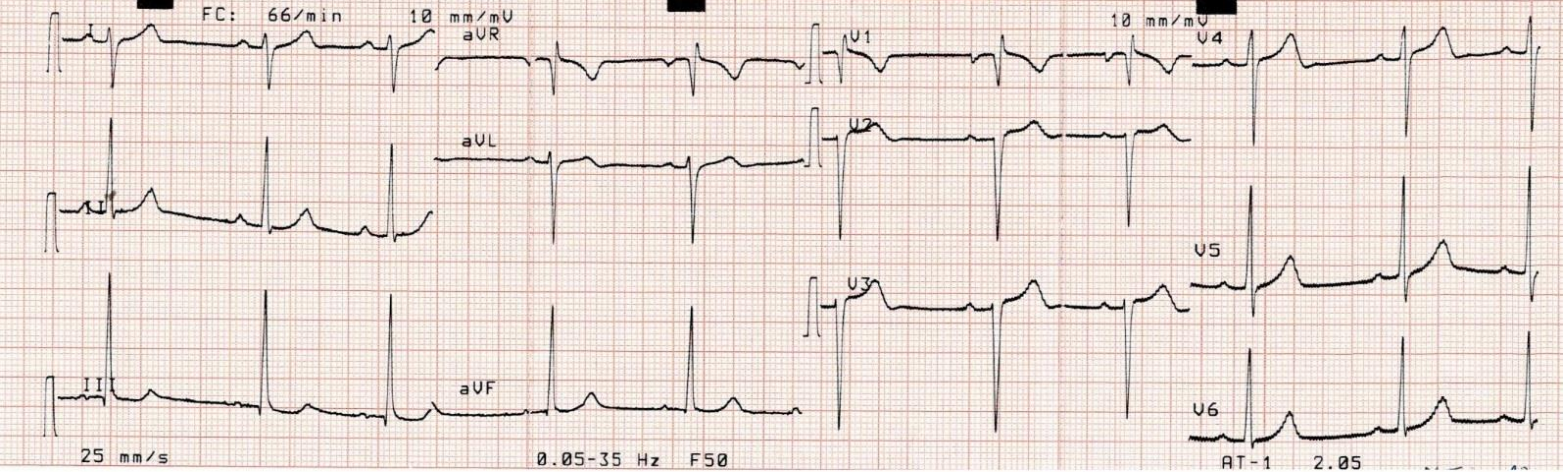
Déviation axiale droite de QRS

Les particularités suivantes: 3 cas (7,5%) d’une onde T négative de V1 à V4 (Figure 1); 1 cas (2,5%) d’une hypertrophie ventriculaire droite (Figure 2); 1 cas (2,5%) de déviation axiale droite de QRS (Figure 3).

Un examen écho-Döppler cardiaque a été effectué chez les 5 sujets présentant des particularités électrocardiographiques. Nous avons relevé une dilatation modérée isolée du ventricule gauche chez un garçon.

## Discussion

L’examen cardiovasculaire avec l’enregistrement systématique d’un ECG sont recommandés dans le cadre de la visite médicale en vue de délivrer un certificat médical de non contre-indication à la pratique de sport en compétition car les causes de mort subite non traumatiques sur les terrains de sport sont dans 85-90% des cas d’origine cardiovasculaire [3-6]. Un effort d’endurance nécessite principalement une augmentation du débit cardiaque. A l’inverse, un effort de résistance mène principalement à une élévation de la pression artérielle qui engendre donc plutôt une surcharge en pression du cœur. Le basket comme la plupart des activités sportives engendre une combinaison d’efforts dynamiques et statiques, si bien que surcharges en volume et pression coexistent le plus souvent. Les athlètes présentent fréquemment des modifications de l’ECG, qui sont essentiellement liées au remodelage ventriculaire et à l’augmentation du tonus vagal [7, 8].

La fréquence cardiaque du sportif est plus basse que celle du sujet non entrainé. Cette constatation est une donnée constante retrouvée par tous les auteurs [7-9]. Les athlètes pratiquant un sport de type isotonique ou sport d’endurance (football, handball, basket) présentent à l’ECG le plus souvent une bradycardie sinusale due à l’hypertonie vagale associée ou non à un bloc auriculo-ventriculaire du 1^er^ ou 2^eme^ degré type Luciani-Wenckebach. Elle ne reflète cependant pas toujours le niveau d’entraînement du sportif [8-10]. Dans notre étude, la fréquence cardiaque au repos plus élevée chez les filles que chez les garçons pourrait être une condition physiologique dont l’explication nécessiterait d’autres investigations. Nous avons observé des atypies électrocardiographiques chez les basketteurs notamment les ondes T négatives en antérieur, la déviation axiale droite de QRS et l’hypertrophie ventriculaire droite.

Des modifications de la repolarisation sont constamment observées chez les sportifs [7-9]. Nous relevons dans notre étude, dans 7,5% des cas (n = 3), une onde T négative de V1 à V4. L’onde T peut être physiologiquement négative dans les précordiales droites en V2 à V3 chez le sujet noir mais habituellement pas au-delà [9]. Cet aspect est certainement attribuable à la pratique intensive de sport chez le sujet noir [9-11]. Nous avons observé un cas de déviation axiale droite de QRS (2,5%) et un cas d’hypertrophie ventriculaire droite (2,5%). L’échographie doppler chez ces patients était normale. Il s’agit d’anomalies non décrites dans la littérature pouvant être une spécificité de l’athlète noir africain [8, 11]. L’exploration d’une atypie ECG chez le sportif doit être prise en charge par un cardiologue et entre alors dans le cadre d’une démarche diagnostique. A ce effet nous avons effectué une échocardiographie Döppler toutes les fois où l’ECG présentait une atypie [4, 10]. Nous avons relevé une dilatation modérée du ventricule gauche chez un garçon basketteur pouvant être attribué dans notre cas à la pratique intensive du basket [7-9].

## Conclusion

La pratique intensive de basket notamment en compétition induit des modifications électrocardiographiques physiologiques chez le jeune noir sénégalais. Toutefois un suivi longitudinal de ces jeunes basketteurs et une étude incluant un plus grand nombre de basketteurs seniors peuvent présenter un grand intérêt.

**Limites de l’étude:** ce travail présente quelques limites dont la petite taille de notre échantillon. Les cas uniques d’atypies électrocardiographiques observés dans notre étude pourraient être confirmés par d’autres études enrôlant un plus grand nombre de sportifs.

### Etat des connaissances actuelles sur le sujet

- Le sport de compétition induit des modifications cardiologiques physiologiques;
- Les modifications du cœur du sportif sont fonctions des disciplines;
- L’ECG du sujet noir peut montrer quelques spécificités.

### Contribution de notre étude à la connaissance

- Les aspects ECG du sujet noir sportif;
- Les aspects ECG du sujet basketteur.

## Conflits des intérêts

Les auteurs ne déclarent aucun conflit d’intérêts.
